# Transcoronary mapping with an over-the-wire multielectrode catheter in scar-related ventricular tachycardia patients

**DOI:** 10.1093/europace/euad365

**Published:** 2023-12-14

**Authors:** Takuro Nishimura, Masahiko Goya, Masateru Takigawa, Miho Negishi, Takashi Ikenouchi, Tasuku Yamamoto, Iwanari Kawamura, Kentaro Goto, Takatoshi Shigeta, Tomomasa Takamiya, Susumu Tao, Taishi Yonetsu, Shinsuke Miyazaki, Tetsuo Sasano

**Affiliations:** Department of Cardiovascular Medicine, Tokyo Medical and Dental University (TMDU), 1-5-45 Yushima, Bunkyo-Ku, Tokyo 113-8510, Japan; Department of Cardiovascular Medicine, Tokyo Medical and Dental University (TMDU), 1-5-45 Yushima, Bunkyo-Ku, Tokyo 113-8510, Japan; Department of Cardiovascular Medicine, Tokyo Medical and Dental University (TMDU), 1-5-45 Yushima, Bunkyo-Ku, Tokyo 113-8510, Japan; Department of Cardiovascular Medicine, Tokyo Medical and Dental University (TMDU), 1-5-45 Yushima, Bunkyo-Ku, Tokyo 113-8510, Japan; Department of Cardiovascular Medicine, Tokyo Medical and Dental University (TMDU), 1-5-45 Yushima, Bunkyo-Ku, Tokyo 113-8510, Japan; Department of Cardiovascular Medicine, Tokyo Medical and Dental University (TMDU), 1-5-45 Yushima, Bunkyo-Ku, Tokyo 113-8510, Japan; Department of Cardiovascular Medicine, Tokyo Medical and Dental University (TMDU), 1-5-45 Yushima, Bunkyo-Ku, Tokyo 113-8510, Japan; Department of Cardiovascular Medicine, Tokyo Medical and Dental University (TMDU), 1-5-45 Yushima, Bunkyo-Ku, Tokyo 113-8510, Japan; Department of Cardiovascular Medicine, Tokyo Medical and Dental University (TMDU), 1-5-45 Yushima, Bunkyo-Ku, Tokyo 113-8510, Japan; Department of Cardiovascular Medicine, Tokyo Medical and Dental University (TMDU), 1-5-45 Yushima, Bunkyo-Ku, Tokyo 113-8510, Japan; Department of Cardiovascular Medicine, Tokyo Medical and Dental University (TMDU), 1-5-45 Yushima, Bunkyo-Ku, Tokyo 113-8510, Japan; Department of Cardiovascular Medicine, Tokyo Medical and Dental University (TMDU), 1-5-45 Yushima, Bunkyo-Ku, Tokyo 113-8510, Japan; Department of Cardiovascular Medicine, Tokyo Medical and Dental University (TMDU), 1-5-45 Yushima, Bunkyo-Ku, Tokyo 113-8510, Japan; Department of Cardiovascular Medicine, Tokyo Medical and Dental University (TMDU), 1-5-45 Yushima, Bunkyo-Ku, Tokyo 113-8510, Japan

**Keywords:** Non-ischaemic cardiomyopathy, Ventricular tachycardia, Coronary artery, High-density mapping

## Abstract

**Aims:**

The usefulness of coronary venous system mapping has been reported for assessing intramural and epicardial substrates in patients with scar-related ventricular tachycardia (VT). However, there has been little data on mapping from coronary arteries. We investigated the safety and utility of mapping from coronary arteries with a novel over-the-wire multielectrode catheter in scar-related VT patients.

**Methods and results:**

Ten consecutive scar-related VT patients with non-ischaemic cardiomyopathy who underwent mapping from a coronary artery were analysed. Six patients underwent simultaneous coronary venous mapping. High-density maps were created by combining the left ventricular endocardium and coronary vessels. Substrate maps were created during the baseline rhythm with 2438 points (IQR 2136–3490 points), including 329 (IQR 59–508 points) in coronary arteries. Abnormal bipolar electrograms were successfully recorded within coronary arteries close to the endocardial substrate in seven patients. During VT, isthmus components were recorded within the coronary vessels in three patients with no discernible isthmus components on endocardial mapping. The ablation terminated the VT from an endocardial site opposite the earliest site in the coronary arteries in five patients.

**Conclusion:**

The transcoronary mapping with an over-the-wire multielectrode catheter can safely record abnormal bipolar electrograms within coronary arteries. Additional mapping data from the coronary vessels have the potential to assess three-dimensional ventricular substrates and circuit structures in scar-related VT patients.

## Graphical Abstract

**Graphical Abstract:**
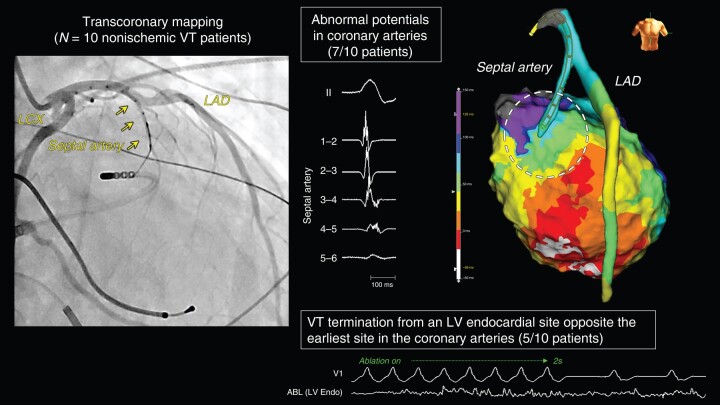

**What's new?**

- A novel over-the-wire microelectrode catheter was safely delivered into the coronary arteries, and abnormal bipolar electrograms were successfully recorded in non-ischaemic scar-related ventricular tachycardia patients.
- Transcoronary mapping can provide additional non-endocardial information after coronary venous system mapping. This strategy might be an alternative to coronary venous system mapping in patients with coronary sinus obstruction.
- High-density substrate map creation combining the coronary vessels and left ventricular endocardium was feasible. The three-dimensional functional substrate and ventricular tachycardia circuit could be assessed by transcoronary mapping.

## Introduction

In non-ischaemic cardiomyopathy (NICM), epicardial and intramural substrates are frequently observed and cause complex reentrant ventricular tachycardia (VT) circuit.^1,2^ Mapping from within the coronary venous system has been reported as a useful technique for detecting these non-endocardial substrates.^3,4^ The development of electroanatomical maps strongly supports assessing the scar-related substrate and VT circuit.^5,6^ Fries *et al.*^7^ successfully delineated coronary arteries with a wire used for an electroanatomical map. In this study, we evaluated the ventricular substrate and VT circuit from the coronary arteries in addition to the left ventricular (LV) endocardium and venous system using an over-the-wire microelectrode catheter.

The aims of this study were to investigate the following:

1. The safety and utility of transcoronary mapping with an over-the-wire microelectrode catheter to assess non-endocardial substrates and the VT circuits in scar-related VT patients.
2. The feasibility of a high-density map creation combining the left ventricular endocardium and coronary arteries.

## Methods

### Patient population

We analysed 10 consecutive NICM patients who underwent mapping within coronary arteries in addition to LV endocardial mapping between 2021 and 2023. The transcoronary mapping was preferentially performed in patients with an obstruction of the coronary venous system or with a potential epicardial adhesion that prevented epicardial mapping with a subxiphoid puncture. The data that support the findings are available from the corresponding author upon reasonable request. The study was approved by the Institutional Review Board of Tokyo Medical and Dental University, and all subjects provided informed and written consent.

### Left ventricular endocardial mapping

All procedures were performed under moderate sedation with dexmedetomidine hydrochloride. Substrate mapping was performed with the EnSite system (Abbott, Abbott Park, IL). During the procedure, heparin was administered so that the ACT stayed within 300–350 s. First, we created LV endocardial high-density maps with a multielectrode catheter (HD Grid Advisor, Abbott) during baseline rhythm. Mapping catheters were inserted through the atrial transseptal approach in all cases. Bipolar voltage mapping was performed using voltage definitions of <0.5 mV for dense scar and <1.5 mV for abnormal voltage areas.^8^ Isochronal late activation maps (ILAMs) were constructed to display the ventricular activation over eight equally distributed isochrones of activation, as previously reported.^5^ Bipolar signals were filtered at 30–500 Hz and displayed at 100 mm/s. We delineated the VT activation map with the window from the QRS onset to the next QRS onset and divided the tachycardia cycle length by eight isochrones (from white to purple). With this method, the QRS onset was represented by the purple–white isochronal interface. The VT isthmus was defined as diastolic activation observed during the middle 50% of the TCL (25–75%).^6^

### Transcoronary mapping

**Figure 1:**
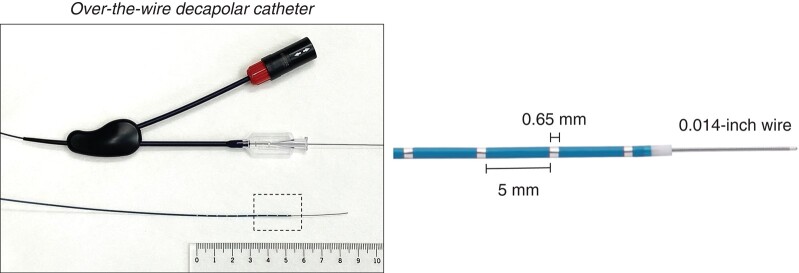
A novel over-the-wire microelectrode catheter. This over-the-wire decapolar catheter was smoothly delivered into the coronary vessels when a 0014 in wire could be inserted into the coronary vessel.

The anatomy of the coronary arterial and venous systems was evaluated by coronary angiography and venography, respectively. Any significant coronary artery stenoses were ruled out. Six patients without coronary venous system occlusion underwent coronary venous system mapping before transcoronary mapping. For mapping the coronary sinus (CS), great cardiac vein (GCV), middle cardiac vein (MCV), and anterior interventricular vein (AIV), a 5Fr decapolar linear catheter or 6F multielectrode catheter with lumen was used. If this was difficult, and when mapping thinner venous branches, a 5Fr angiographic catheter (Judkins right) was used to engage the CS from the femoral vein and to advance a 0.014 inch wire to the target vessel. This wire was then used to guide a microelectrode catheter into the target vessel. A novel over-the-wire catheter [EPstar Fix AIV (2.7 Fr); Japan Lifeline, Tokyo, 0.65 mm ring, 5 mm edge-to-edge spacing, *Figure 1*] was used.

We performed mapping from the arterial system to assess more non-endocardial substrates. Transcoronary mapping was performed preferentially in the branch of the coronary vessel adjacent to the endocardial deceleration zone (DZ) or late gadolinium enhancement region detected by cardiac MRI. If there were appropriate arterial branches, a 6Fr guiding catheter was deployed in the coronary artery, and a 0.014 inch guidewire was inserted into the target arterial branch as distally as possible to obtain a sufficient backup force. An over-the-wire microelectrode catheter was then advanced to the target site, keeping careful watch for ST segment changes. The ACT was checked every 15 min and controlled within 350–400 s while the over-the-wire catheter was inserted within the coronary artery.

### Radiofrequency ablation

Endocardial substrate modification was performed within the abnormal voltage area guided by matched pacemaps or DZs for complete non-inducibility, for the procedural endpoint. When targeting a non-endocardial isthmus component detected by transcoronary mapping, radiofrequency ablation was performed at the endocardial site as anatomically close as possible to the earliest activation site recorded within the coronary vessel. When radiofrequency energy was delivered, the over-the-wire catheter was pulled out of the coronary artery. Ablation was performed with an open-irrigated catheter (TactiCath or FlexAbility, Abbott) with 30–50 W and a temperature limit of 45°C with a 13–30 mL/min flow rate. We did not remap the DZ after ablation and did not assess whether pacing could capture the myocardium after ablation in this study. The VT inducibility after ablation was assessed by burst pacing (600 ms sequentially down to 300 ms) and programmed extrastimuli with a drive train of 400–600 ms down to triple extrastimuli to the ventricular effective refractory period or 200 ms.

After the ablation procedure, we performed coronary angiography to assess the status of the coronary arteries in all cases. The changes in the ST segment were evaluated by a 12-lead electrocardiogram during the procedure, immediately after returning to the ward and the day after the procedure.

### Statistical analysis

Continuous variables are expressed as the median and interquartile range. Categorical variables are presented as percentages. Statistical analyses were conducted using JMP software (version 12.2.0, SAS Institute Inc., Cary, NC, USA).

## Results

The characteristics of the 10 patients who underwent transcoronary mapping are summarized in *Table 1*. The median left ventricular ejection fraction was 34% (IQR 24–42%). All but one patient had a history of a failed catheter ablation. Half of the patients had potential epicardial adhesions due to prior cardiac surgery (*n* = 2) or a prior failed epicardial ablation (*n* = 3). In four patients, the coronary venous system was occluded due to adhesion of the LV lead for cardiac resynchronization therapy (*n* = 3) or due to a dissection when an unsuccessful insertion of the LV lead occurred (*n* = 1). Four patients were defined as having an anteroseptal substrate, and the remaining six patients were categorized as having an inferolateral substrate, according to the previously reported classification.^9,10^

**Table 1:** Characteristics and mapping vasculatures

Patient	Age	Sex	LVEF (%)	Subtype	Risk of epicardial adhesions	CS occlusion	Mapping vasculatures	
							Coronary venous system	Coronary arteries
1	68	M	34	AS	Post-epi ablation	0	CS, GCV, AIV	LAD, D1, septal artery
2	79	M	45	IL	Post-AVR	0	CS, GCV	LCX
3	55	M	35	IL	Post-epi ablation	1		LAD, LCX
4	67	M	41	IL	None	0	CS, GCV, AIV, MCV, lateral vein	LCX
5	55	M	18	AS	None	1		LAD septal artery
6	65	M	43	AS	Post-epi ablation	0	CS, GCV, AIV, septal perforator	LAD, D1, LCX
7	77	M	32	AS	None	0	CS, GCV, AIV	LAD, LCX
8	45	F	20	IL	Post-ASD closure	1		LAD, LCX
9	59	M	25	IL	None	1		LCX
10	67	F	34	IL	None	0	CS, GCV, AIV, MCV	RCA

AIV, anterior interventricular vein; AS, anteroseptal substrate; AVR, aortic valve replacement; ASD, atrial septal defect; CS, coronary sinus; D1, the first diagonal branch of LAD; GCV, great cardiac vein; IL, inferolateral substrate; LCX, left circumflex artery; LAD, left anterior descending artery; MCV, middle cardiac vein.

### Transcoronary substrate mapping

Substrate maps during baseline rhythm were created with 2438 points (IQR 2136–3490 points). The median mapping points in the venous system were 234 points (IQR 52–626 points). The ILAM revealed an endocardial DZ in seven patients. Among the six patients who underwent coronary venous mapping, fragmented potentials and late potentials were recorded in two patients each.

**Figure 2:**
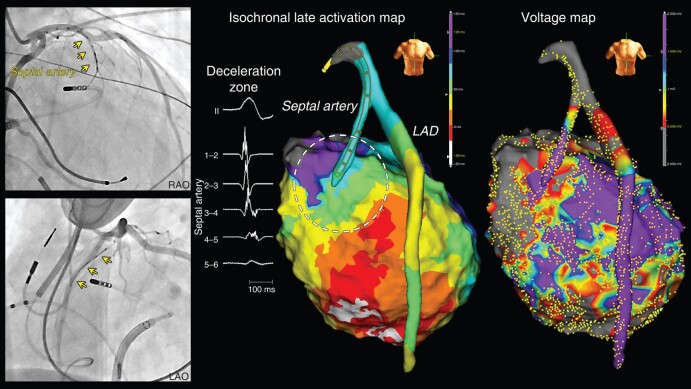
Transcoronary mapping in a septal artery. A patient with a CS occlusion due to an adhesion of an LV lead (Patient 5). Fragmented potentials were recorded in the septal artery during sinus rhythm. The ILAM exhibited a periaortic deceleration zone (dot circle). An abnormal voltage area was detected in the proximal LAD and basal anteroseptal region on the left ventricular endocardium. CS, coronary sinus; ILAM, isochronal late activation map; LAD, left anterior descending artery; LV, left ventricle.

**Figure 3:**
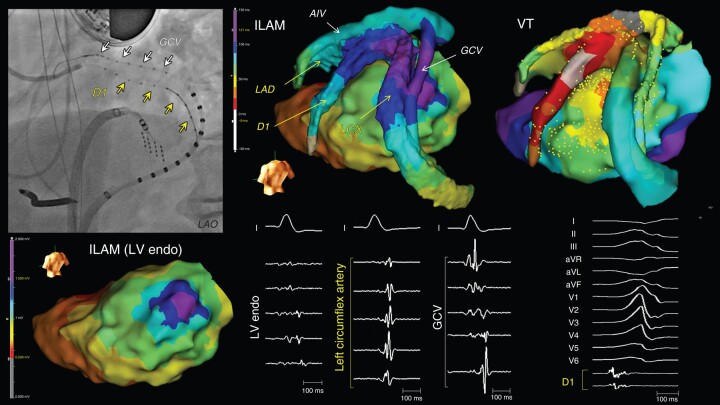
Transmural substrate and the earliest VT activation site recorded in the first diagonal branch of LAD (Patient 6). The over-the-wire catheters were simultaneously inserted into the GCV and the first diagonal branch of LAD. Late potentials were recorded on the LV endocardium, coronary venous system, and coronary arteries, suggesting transmural substrate. The earliest VT activation (−36 ms from QRS onset) was recorded in the first diagonal branch. The VT was successfully terminated by endocardial ablation at the opposite site of the first diagonal branch. AIV, anterior interventricular vein; D1, the first diagonal branch; GCV, great cardiac vein; ILAM, isochronal late activation map; LAD, left anterior descending artery; LCX, left circumflex artery; LV, left ventricle.

**Figure 4:**
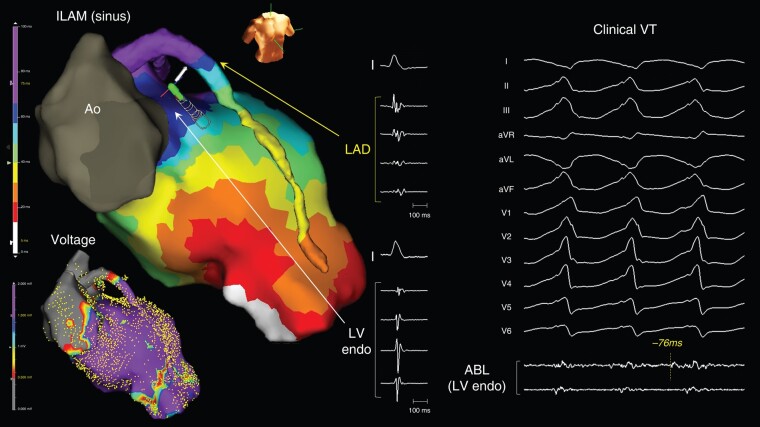
Deceleration zone recorded in the left anterior descending artery. A patient with a CS occlusion with an LV lead (Patient 7). A deceleration zone with fragmented potentials was recorded in the LAD, but there were no abnormal potentials on the LV endocardium. During the VT, far-field pre-systolic potentials (−76 ms preceding QRS onset) were recorded from the LV endocardium, and the VT was terminated by endocardial ablation. Ao, aorta; CS, coronary sinus; LAD, left anterior descending artery.

**Figure 5:**
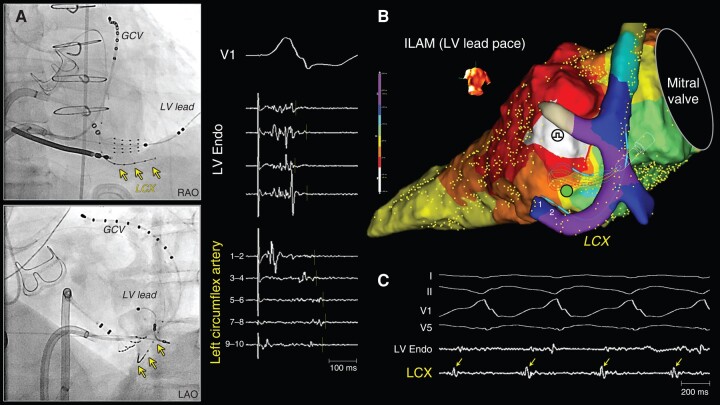
Late potentials recording in the left circumflex artery and successful VT ablation targeting mid-diastolic potentials recorded in the coronary artery. A patient with a prior aortic valve replacement (Patient 2). (*A*) The over-the-wire microelectrode catheter was inserted into the LCX. A grid catheter was placed on the basal lateral endocardium. Fractionated electrograms were recorded on the endocardium, but late potentials were only detected within the LCX. (*B*) An ILAM during pacing from the LV lead of a CRT-D device. The latest activation was recorded in the LCX; however, the pacing was performed from the CS, which was adjacent to the LCX on the epicardium. This phenomenon suggested that there was a transmural conduction block and an intramural conduction barrier (Supplementary material online, *Movie*). (*C*) During the VT, mid-diastolic potentials were recorded in the LCX (arrows). The ablation from an endocardial site opposite the LCX terminated the VT (dot). GCV, great cardiac vein; ILAM, isochronal late activation map; LCX, left circumflex artery; RF, radiofrequency ablation.

Mapping in the coronary artery was successfully performed in all cases with 329 points (IQR 59–508 points). The left anterior descending artery (LAD, *Figures 2, 3,* and *4*) and left circumflex artery (LCX, *Figures 3, 5A,* and *6*) were mapped in six and seven patients, respectively. The septal artery and the first diagonal branch of LAD were mapped in two patients each (*Figures 2* and *3*). Mapping was performed in the large right coronary artery of one patient to assess the inferior region. Abnormal electrograms were recorded within the coronary arteries in seven patients during the baseline rhythm. Fragmented potentials (*Figures 2* and *4*) and late potentials (*Figures 3* and *5A*) were recorded in three and four patients, respectively.

In Patient 2 with a history of an aortic valve replacement, a three-dimensional (3D) substrate was suspected from the results of the high-density endocardial and the transcoronary maps. The latest activation was recorded on the over-the-wire catheter in the LCX while the pacing was performed from the lateral vein [the cardiac resynchronization therapy defibrillator (CRT-D) LV lead], which was adjacent to the LCX on the epicardium. This phenomenon suggested that there was a transmural conduction block and an intramural conduction barrier (*Figure 5B*, Supplementary material online, *Movie*).

### Transcoronary ventricular tachycardia mapping and ablation

**Figure 6:**
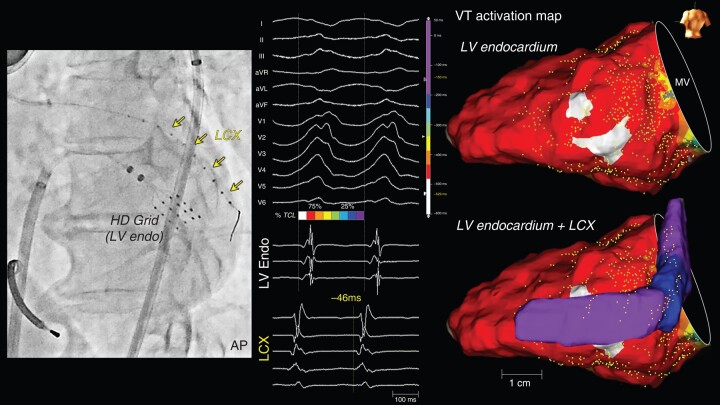
A focal breakout VT with pre-systolic potentials recorded in the left circumflex artery. A patient with a CS occlusion due to an unsuccessful LV lead insertion (Patient 3). An over-the-wire catheter was placed in the middle portion of the LCX. A grid catheter was placed on the basal lateral endocardium. During the VT, pre-systolic electrograms preceding the QRS onset by 46 ms were recorded within the LCX. The endocardial VT activation map exhibited a focal breakout pattern from two sites. In LCX, pre-systolic electrograms were recorded 2.5 cm away from the endocardial breakout sites. CS, coronary sinus; LCX, left circumflex artery; LV, left ventricle; MV, mitral valve.

Monomorphic VT was induced during the procedures in eight patients. During VT, mid-diastolic potentials were recorded within the LCX in one patient (*Figure 5C*). Pre-systolic potentials were recorded in three patients (LAD: *n* = 2, LCX: *n* = 1) (*Figures 4* and *6*). In three patients, the isthmus components were recorded within the coronary vessels without any discernible isthmus components on the endocardial mapping.

In Patient 3 with a CS occlusion due to a dissection from an unsuccessful insertion of an LV lead (*Figure 6*), transcoronary mapping was useful to detect epicardial focal VTs. During the VT, the endocardial activation map could not detect any potentials earlier than the onset of the QRS. Transcoronary mapping demonstrated pre-systolic electrograms preceding the QRS onset by 46 ms that were recorded within the LCX. This pre-systolic electrograms were recorded 2.5 cm away from the endocardial focal breakout site. The ablation terminated the VT at an endocardial site opposite the LCX.

VT termination was achieved while ablation at the closest endocardial site opposite the target arteries in five patients (LAD: *n* = 2, LCX: *n* = 2, the first diagonal branch of LAD: *n* = 1). The abnormal electrograms recorded within the coronary arteries were not eliminated but were modified after the ablation at the endocardial closest site in all cases. The final lesion set was created by localized homogenization of the endocardial DZ and intensive ablation at the endocardial site close to the isthmus component recorded within the coronary vessels. At the end of the procedure, non-inducibility was confirmed in five patients. Non-clinical VT remained in two patients. We did not assess the inducibility in three patients by discretion of the operators.

In the post-procedural coronary angiography, there was no injury or stenosis observed within the coronary arteries in any of the cases. No ST segment changes were observed during the procedures, immediately after returning to the ward, and the day after the procedures in any of the cases. No groin complication was observed. There were no complications related to the transcoronary mapping and ablation.

## Discussion

The major findings of the present study were as follows:

1. A novel over-the-wire microelectrode catheter successfully recorded the abnormal bipolar electrograms within the coronary arteries in scar-related VT patients.
2. High-density delineation combining the coronary artery and LV endocardium was feasible. The 3D functional substrate and VT circuit could be assessed by transcoronary mapping.

Coronary venous system mapping with a microelectrode catheter has been reported previously for ventricular arrhythmias.^11–13^ We successfully applied this method to map coronary arteries in scar-related VT patients. Accessibility to the coronary vasculature by a microelectrode catheter is critical for a high-density substrate evaluation. The novel over-the-wire catheter has the potential to approach the target vessels safely as compared with that without an over-the-wire option. There are a few reports of mapping within the arterial side of the coronary vasculature in scar-related VT patients. Tholakanahalli *et al.*^14^ reported two cases with intracoronary wire mapping and coil embolization for an intraventricular septal substrate. Kataoka *et al.*^15^ assessed the bipolar electrograms within the LCX and treated a challenging scar-related VT case by an ethanol infusion. Recently, Fries *et al.*^7^ reported the feasibility of visualizing the coronary arteries on the electroanatomical map by wire mapping. To the best of our knowledge, this is the first report to assess the abnormal bipolar electrograms from coronary arteries and to create a high-density transcoronary map.

A CS obstruction can occur due to a prior cardiac surgery or previous CRT implantation.^16^ Transcoronary mapping may be an alternative option for evaluating non-endocardial substrates in patients with CS obstruction. Although epicardial mapping and ablation of NICM are broadly used and an subxiphoid puncture is an established technique, it is not without risk.^17–19^ Epicardial adhesions can make epicardial mapping challenging and more invasive, requiring a surgical access.^20^ Mapping and ablation from the coronary venous system has already been reported as a useful option in post-coronary bypass patients.^21^ It might be reasonable to attempt transcoronary mapping in addition to coronary venous mapping before proceeding to an epicardial approach with a possible epicardial adhesion.

Endocardial radiofrequency ablation targeting the VT isthmus recorded within the coronary artery terminated the VT; however, it failed to completely eliminate the late potentials within the coronary artery. There were some potential reasons. First, the cooling effect from the blood flow in the coronary arteries might limit the lesion creation. Second, the electrograms recorded within the coronary artery can be formed by activation from multiple directions, and radiofrequency ablation from one direction may have an insufficient effect to eliminate the abnormal electrograms with the coronary arteries.

A VT circuit with a completely intramural isthmus presents a focal breakout pattern on both the endocardium and epicardium. In such cases, a common strategy might be localized homogenization around the focal activation site on both cardiac surfaces. However, the exit and mid-isthmus can be spatially separated in the reentrant circuit.^6^ High-density transcoronary and venous system mapping have the potential to reveal an intramural functional substrate and intramural components of a 3D VT, adding information to that gained by simultaneous endocardial and epicardial mapping. The final lesion set could be changed when the isthmus component was detected by transcoronary mapping. Further studies to evaluate pacemapping and entrainment mapping from the coronary vessels to identify the specific isthmus component are desired.^22,23^

### Limitations

This was a single-centre analysis with a limited number of cases. Transcoronary mapping may underestimate the prevalence of a substrate, which can be evaluated only in areas that lie under accessible branches of the coronary vessels. Identifying the conducting channel using cardiac magnetic resonance imaging has recently been reported; however, the present study did not include such a detailed assessment of the cardiac imaging.^24^ We did not assess the efficacy of the ablation at the best endocardial mapping site when the isthmus component was recorded only within the coronary vessels. We did not evaluate the safety of this procedure in patients with ischaemic cardiomyopathy.

## Conclusion

The transcoronary mapping with an over-the-wire multielectrode catheter can safely record the abnormal bipolar potentials within coronary arteries. Additional mapping data from the coronary vessels have a potential to assess 3D ventricular substrates and/or circuit structures in scar-related VT patients.

## Supplementary Material

euad365_Supplementary_Data

## Data Availability

The data that support the findings of this study are available from the corresponding author upon reasonable request.
